# First-in-Human Phase I Study of Minnelide in Patients With Advanced Gastrointestinal Cancers: Safety, Pharmacokinetics, Pharmacodynamics, and Antitumor Activity

**DOI:** 10.1093/oncolo/oyad278

**Published:** 2024-01-03

**Authors:** Erkut Borazanci, Ashok Saluja, Jon Gockerman, Mohana Velagapudi, Ronald Korn, Daniel Von Hoff, Ed Greeno

**Affiliations:** HonorHealth Research Institute, Scottsdale, AZ, USA; Translational Genomics Research Institute (TGen), Phoenix, AZ, USA; Minneamrita, Tampa, FL, USA; Novella Clinical, Morrisville, NC, USA; Minneamrita, Tampa, FL, USA; HonorHealth Research Institute, Scottsdale, AZ, USA; Imaging Endpoints, Scottsdale, AZ, USA; HonorHealth Research Institute, Scottsdale, AZ, USA; Translational Genomics Research Institute (TGen), Phoenix, AZ, USA; Masonic Cancer Center, Minneapolis, MI, USA

**Keywords:** Minnelide, triptolide, gastrointestinal cancer, pancreatic cancer, gastric cancer

## Abstract

**Background:**

Minnelide is a water-soluble prodrug of triptolide. Triptolide is an anticancer agent that targets cancer resistance through several mechanisms. Minnelide was evaluated in a phase I study in patients with advanced GI carcinomas to establish the safety, pharmacodynamic, antitumor activity, and recommended phase II dose (RP2D).

**Patients and Methods:**

Patients with refractory GI carcinoma and with measurable disease on CT scan were eligible. The study used a 3 + 3 dose-escalation scheme. Due to neutropenia toxicity, 2 dosing schedules were evaluated to determine the RP2D for future studies. Response was assessed using RECIST 1.1 and Choi criteria. Minnelide and triptolide PK were evaluated. Patients who completed the first 28-day treatment cycle without DLTs continued treatment until disease progression or unacceptable toxicity.

**Results:**

Forty-five patients were enrolled (23 pancreatic cancer, 10 colorectal, and the remaining 9 had other GI tumors); 42 patients received at least one dose of Minnelide. Grade ≥ 3 toxicities occurred in 69% of patients, most common neutropenia (38%). 2 patients with severe cerebellar toxicity who had a 2-fold higher triptolide concentration than other participants. ORR was 4%; the disease control rate (DCR) was 54% (15/28). Choi criteria demonstrated a decrease in average tumor density in 57% (16/28) patients.

**Conclusions:**

This first-in-human, phase I clinical study identified a dose and schedule of Minnelide in patients with refractory GI cancers. The primary toxicity experienced was hematologic. Evidence of efficacy of Minnelide treatment in this group of patients was observed. The DCR ranged from ~2 to 6 months in 14/28 (50%) of evaluable patients. Studies in monotherapy and combination treatments are underway.

## Teaser

In this phase I study, minnelide was evaluated in patients with advanced gastrointestinal carcinomas to establish the safety, pharmacodynamic, antitumor activity, and recommended phase II dose.

**Implications for Practice**

While significant progress has been made to treat gastrointestinal (GI) cancers, there remains a need to look for additional therapeutic options. Minnelide is a novel compound that has been found to target HSP70 along with apoptosis, EMT, and transcription in malignancies. In a phase I study for refractory GI cancers Minnelide showed evidence of clinical benefit and is currently being explored in several clinical trials in oral formulation, both in single agent and in combination with other chemotherapeutic agents.

## Introduction

Gastrointestinal (GI) tumors will account for over 169 000 deaths in the US in 2021,^1^ including >43 000 deaths from pancreatic cancer [Evan 2017]. Despite recent advances with systemic therapy, options remain limited upon resistance to chemotherapy. Several hallmarks of cancer contribute to chemotherapy resistance including cell death by apoptosis; autophagy; reliance on the epithelial-mesencyhmal transition (EMT) to invade, metastasize, and resist apoptosis; immune system modulation; and activation of key transcriptional pathways for growth [Hanahan 2011; Evan 2017]. New therapeutics are needed to improve survival rates for patients with metastatic gastrointestinal (GI) tumors.

Triptolide is a diterpenoid triepoxide found in the Chinese plant *Tripterygium wilfordii*, which has been used as a natural medicine in China for hundreds of years, particularly in autoimmune and inflammatory diseases.^2^ Triptolide also targets cancer resistance through several mechanisms, including induction of apoptosis.^2^ This has been shown in animal cancer models, including cholangiocarcinoma^3^ and xenograft tumor models including melanoma, breast cancer, bladder cancer, gastric carcinoma,^4^ and pancreatic cancers.^5^ Some of triptolide’s effect is via suppression of heat shock protein (HSP)70, which inhibits cell death and is one mechanism of tumor resistance. While the mechanism of triptolide inhibition of HSP70 expression is not fully understood, it has been shown to induce caspase activation.^6-9^ In the MIA-PaCa2 and PANC-1 pancreatic cancer cell lines, cytochrome *c* release from mitochondria and subsequent caspase-3 activity increased after incubation with triptolide, suggesting that the mitochondrial apoptotic pathway is responsible for triptolide-induced cell death.^5^

Triptolide also induces autophagic cell death in some tumor types (metastatic lines S2-013, S2-VP10, and Hs766T) associated with inactivation of the protein kinase B (Akt)/mammalian target of rapamycin/p70S6K pathway and upregulation of the extracellular signal-related kinase 1/2 pathway. Triptolide inhibits EMT in pancreatic cancer cells through downregulation of NF-kB signaling, which leads to the downregulation of several mesenchymal factors.^10^ Additional effects on EMT by triptolide have been reported in breast, ovarian, and liver cancer models.^11-13^ Triptolide has been implicated in modulating transcription by depleting RPB1, the largest subunit of RNA polymerase II in non-small cell lung cancer cells.^14^ In pancreatic ductal adenocarcinoma, triptolide has been reported to downregulate c-MYC, a major driver of oncogenesis through super-enhancer programs,^15^ in both stroma and cancer cell compartments.^16^

Triptolide is poorly soluble in water; therefore, a water-soluble analog of triptolide, Minnelide (14-O-phosphonooxymethyltriptolide disodium salt) was synthesized. Minnelide is a prodrug that rapidly releases triptolide when exposed to phosphatases in the bloodstream. The efficacy of Minnelide was tested both in vitro and in multiple in vivo models of pancreatic cancer: an orthotopic model of human pancreatic cancer cells in athymic nude mice, a xenograft model of primary human pancreatic tumors transplanted into severe combined immunodeficient mice, and a spontaneous pancreatic cancer mouse model (KRasG12D, Trp53R172H, Pdx-1Cre). In these models of pancreatic cancer, Minnelide reduced pancreatic tumor growth and spread, and improved survival.^17,18^ Other GI tumor model systems have shown sensitivity to Minnelide, including colorectal cancer.^19^

The antitumor activity of Minnelide reduced tumor volumes and markedly improved survival rates in animals suggests that Minnelide may improve outcomes in patients with GI cancers. Therefore, we conducted a first-in-human phase I trial of Minnelide to evaluate its safety, pharmacokinetics (PK), and pharmacodynamics in patients with advanced GI malignancies who progressed on standard therapy.

## Methods

This was a phase I, open-label, multicenter, dose-escalation study of Minnelide in adults with advanced GI carcinoma (NCT01927965). The study sites included HonorHealth Research Institute (Scottsdale, AZ) and University of Minnesota (Minneapolis, MN). This study was performed in accordance with Good Clinical Practice, and all patients provided signed informed consent prior to study enrollment. The study protocol and informed consent form were reviewed and approved by the appropriate Institutional Review Board/Ethics Committee before the start of the study.

### Patient Eligibility

Patients with histologically or cytologically confirmed GI carcinoma, which had progressed on standard therapies (surgery, radiotherapy, endocrine therapy, chemotherapy), for whom effective therapy was not available and who had at least one lesion measurable on computed tomography (CT) scan, were eligible for the study. Other eligibility criteria included: age ≥18 years; Karnofsky performance status of >70% with a life expectancy of >3 months; adequate bone marrow, liver, and renal function; and ability to provide informed consent. Patients were excluded from the study if they had received chemotherapy, radiotherapy, or investigational therapy <1 month before enrollment. Patients were excluded for pregnancy, significant heart disease, baseline QTc>450 msec (470 msec for females) using Bazett’s formula, uncontrolled infection, known human immunodeficiency virus (HIV) or viral hepatitis, or other serious comorbidities.

### Study Design and Treatment

The primary objectives of this study were to determine the maximum tolerated dose (MTD) and dose-limiting toxicities (DLTs) of Minnelide and establish the recommended dose for future studies. The secondary objectives of this study were to evaluate the PK of Minnelide, observe patients for evidence of antitumor activity, determine the pharmacodynamic effect on HSP70 levels, and explore the pharmacodynamic effects on imaging.

The study used a standard 3 + 3 dose-escalation scheme with the starting dose of 0.16 mg/m^2^ daily, based on toxicology data in animals. Initially, patients were administered Minnelide daily for 21 days in a 28-day cycle (dosing schedule A). Following Protocol Amendment 2, a second dosing schedule of Minnelide administered for 5 of every 7 days for the first 21 days of a 28-day cycle was introduced (dosing schedule B). The change was introduced to help prevent neutropenia, which was observed during schedule A. Minnelide was administered once per day as a 30-minute intravenous infusion at the dose and schedule prescribed by the escalation schema.

Three to 6 patients were enrolled in each dose group using schedule A or schedule B. Twenty-seven patients were enrolled in schedule A, with dose levels 0.16 mg/m^2^ (3 patients), 0.32 mg/m^2^ (3 patients), 0.53 mg/m^2^ (12 patients), 0.67 mg/m^2^ (6 patients), and 0.8 mg/m^2^ (3 patients). Fifteen patients were enrolled in schedule B, with dose levels 0.67 mg/m^2^ (5 patients), 0.80 mg/m^2^ (6 patients), and 1.00 mg/m^2^ (4 patients).

Patients who completed the first 28-day treatment cycle without significant treatment-related toxicities or clinical evidence of progressive disease were allowed to continue treatment in 28-day cycles until evidence of disease progression (defined by Response Evaluation Criteria in Solid Tumors [RECIST] v 1.1 criteria) or unacceptable toxicity.

### Safety Monitoring

Safety was monitored from the first dose of study drug, throughout the study, and for 30 days after the last dose of study drug. Adverse events (AEs), clinical laboratory tests, vital signs, physical examination, and electrocardiograms (ECGs) were evaluated. Neurological examinations were performed weekly for the first 6 weeks, followed by once each cycle to check for dysarthria, dysdiadochokinesia, and gait and truncal ataxia. Study staff questioned patients each day regarding neurological status. Complete blood count with differential was performed daily during cycle 1 and as clinically indicated but at least twice weekly for cycle 2 and beyond.

AEs were documented by body system and preferred term according to Medical Dictionary for Regulatory Activities (MedDRA) terminology, and AE intensity (severity) was graded according to the NCI Common Terminology Criteria for AEs (CTCAE) v4.0. AE relationship to study drug was evaluated by the investigator.

A DLT was defined as any of the following: grade 4 neutropenia lasting ≥5 days or grade 3 or 4 neutropenia with fever and/or infection; grade 4 thrombocytopenia (or Grade 3 with bleeding); grade 3 or 4 treatment-related non-hematological toxicity (grade 3 nausea, vomiting, or diarrhea that lasted >72 hours, despite maximal treatment constituted a DLT); dosing delay greater than 2 weeks due to treatment-emergent adverse events (TEAEs) or related severe laboratory abnormalities. Patients could have one dose reduction for toxicity.

The MTD is the dose at which no more than a third of the patients experienced a DLT during the first cycle.

### Study Assessments

Baseline data collection included a complete history, physical and neurological exams, ECG, clinical chemistries, blood counts, coagulation studies, urinalysis, HSP70 blood levels, clinically relevant tumor markers, and a positron emission tomography (PET)/CT scan. Routine laboratory studies were repeated twice the first week and then weekly thereafter.

#### Pharmacokinetics

Blood was collected for plasma from each patient at approximately 0.25 hour from the start of infusion, the end of infusion, and approximately 5 minutes, 0.25, 0.5 1, 2, 4, 6, and 8 hours following the end of the infusion on day 1 and day 15 for both schedules A and B. Predose samples were collected on days 1, 8, and 15. An additional sample was collected from schedule A patients on day 22. Samples were analyzed using validated methods for Minnelide and its active metabolite, triptolide, at Charles River Laboratories (Senneville, QC, Canada). Minnelide and triptolide plasma concentrations versus actual sample time for each patient were analyzed by noncompartmental analyses (intravenous [IV] infusion) using a validated installation of Phoenix WinNolin Version 6.4 (Certara Corporation, Princeton, NJ). Values below the lower limit of quantitation were treated as missing.

#### Disease Monitoring

To objectively measure tumor size, CT and PET scans were obtained at baseline, PET scans at day 22 of each cycle, and CT scans at the end of cycles 2, 4, 6, and every 3 cycles thereafter. Imaging standardization, collection, and central review were performed by core laboratory Imaging Endpoints, LLC (Scottsdale, Arizona). RECIST v1.1 criteria were used to assess tumor activity.^20^ Exploratory imaging analyses for tumor density changes utilizing Choi criteria as a measure of tumor necrosis (ie, apoptosis)^21^ and for tumor metabolism utilizing European Organization for Research and Treatment of Cancer (EORTC) criteria.

Efficacy was analyzed by objective response rate (ORR), best overall response (BOR), and progression-free survival (PFS). ORR was determined from the number of patients experiencing a complete response (CR) or partial response (PR). BOR was the best response recorded from the start of the treatment until the end of study, categorized as CR, PR, Stable Disease (SD), Progressive Disease (PD), and Unable to Evaluate or Not Applicable. PFS was defined as the number of days from the first treatment to the earliest date of progression or death from any cause. If the patient did not progress or die, the PFS time was censored at the last disease assessment.

#### Pharmacodynamics

Pharmacodynamics assessments performed included: serum HSP70 levels; PET scans; CT scans using Choi criteria; and biomarker analyses including CA19-9 (or CEA and CA125 as applicable). HSP70 levels and tumor biomarkers were collected on day 1 of each cycle. PET and CT scans were obtained as described above. Serum HSP70 level was quantitated by ELISA (ENZO Life Sciences [cat # #KS-700B]). Imaging was assessed using RECIST 1.1 Criteria. PET Scans (SUV_max_) and CT scans (Choi Criteria) also were evaluated for pharmacodynamic effect.

### Statistical Analysis

The study population used in analyses included patients who received any amount of study drug. Data were summarized according to dose groups. Up to 54 patients were planned for enrollment in the study. If a patient discontinued or was withdrawn during cycle 1 unrelated to toxicity, the patient may have been replaced. The MTD was determined following a traditional 3 + 3 design. For ORR, exact 95% CIs were constructed using exact methods for the binomial distribution. Safety, efficacy, and PK data were summarized using descriptive statistics. Summary statistics for Minnelide and triptolide plasma concentrations and PK parameter estimates were generated by Novella Clinical using SAS (SAS Institute, Cary, NC).

## Results

### Patient Characteristics

Forty-five patients were enrolled between September 2013 and June 2016. Forty-two patients received at least 1 dose of drug and were analyzed, 27 on schedule A and 15 on schedule B. Of the 42 patients, 23 had pancreatic cancer, 10 colorectal, and the remaining 9 had other GI tumors. All had a least 1 prior chemotherapy regimen, with more than half receiving 3 or more regimens. Table 1 has patient demographics and baseline characteristics.

**Table 1.** Demographics and disease characteristics of patients receiving at least 1 dose of Minnelide.

Characteristic	Schedule A (*N* = 27)	Schedule B(*N* = 15)	All patients (*N* = 42)
Gender, *n* (%)			
Male	9 (33)	7 (47)	16 (38)
Female	18 (67)	8 (53)	26 (62)
Age, years			
Median	60.0	60.0	60
Min, max	37, 75	36, 82	36, 82
Race/ethnicity, *n* (%)			
White, non-Hispanic	27 (100)	14 (93)	41 (98)
White, Hispanic	2 (7)	0 (0)	2 (5)
Asian	0 (0)	1 (7)	1 (2)
Prior therapy, *n* (%)			
Chemotherapy	27 (100)	15 (100)	42 (100)
Regimen group number^a^			
1-2 regimen(s)	69 (43)	53 (55)	122 (47)
≥3 regimens	92 (57)	44 (45)	136 (53)
Tumor types, *n* (%)			
Pancreas	16 (59)	7 (47)	23 (55)
Colorectal	8 (30)	2 (13)	10 (24)
Other gastrointestinal	3 (11)^b^	6 (40)^c^	9 (21)

^a^Percentage based on the total number of therapies for each treatment group or overall as appropriate.

^b^Includes 1 gastric, 1 hepatocellular, and 1 other primary diagnosis.

^c^Includes 1 esophageal, 1 gastric, and 4 other primary diagnosis.

### Safety

Forty-two patients received at least one dose of drug, with a median of 2 cycles and a maximum of 8 cycles. Table 2 summarizes the AEs related to Minnelide. These AEs were reported in 28 patients (67%) for hematologic disorders, 15 patients (36%) for gastrointestinal disorders, 12 patients (29%) for general disorders, and 4 patients (10%) each for metabolic and neurological disorders. Grade 3 or greater AEs occurred in 69% (29/42) of patients, with 43% (18/42) attributed to study drug. The most common grade 3 or higher TEAE was neutropenia (16 patients, 38%).

**Table 2.** Treatment-emergent adverse events (TEAEs) in patients receiving at least 1 dose of Minnelide.

	Related to Study Drug			
	Any TEAE	Grade 3, 4, or 5 TEAE	Grade 3, 4, or 5 TEAE	Dose-limiting toxicity
Treatment schedule, *n* (%)				
Schedule A (*n* = 27)	24 (89)	13 (48)	19 (70)	3 (11)^a^
Schedule B (*n* = 15)	8 (53)	5 (33)	10 (67)	1 (7)^b^
Both schedules (*n* = 42), n (%)				
Blood/lymphatic	28 (67)	N/A	21 (50)	3 (7)
Neutropenia	19 (45)	N/A	16 (38)	1 (2)
Thrombocytopenia	17 (41)	N/A	6 (14)	1 (2)
Anemia	12 (29)	N/A	6 (14)	0 (0)
Leukopenia	10 (24)	N/A	5 (12)	0 (0)
Lymphopenia	6 (14)	N/A	2 (5)	0 (0)
Coagulopathy	2 (5)	N/A	0 (0)	0 (0)
Neutropenic fever	0 (0)	N/A	2 (5)	1 (2)
Gastrointestinal	15 (36)	N/A	5 (12)	0 (0)
General disorders	12 (29)	N/A	0 (0)	0 (0)
Fatigue	9 (21)	N/A	0 (0)	0 (0)
Metabolic	4 (10)	N/A	7 (17)	0 (0)
Neurological	4 (10)	N/A	2 (5)	2 (5)
Hepatobiliary	3 (7)	N/A	5 (12)	0 (0)
Infection	2 (5)	N/A	4 (10)	0 (0)

^a^Two patients were on 0.67 mg/m^2^ dose and one patient was on 0.80 mg/m^2^ dose.

^b^Patient dose was 1.00 mg/m^2^.

Three of 27 patients on schedule A (2/6 patients on 0.67 mg/m^2^ dose and 1/3 patients on 0.80 mg/m^2^ dose) experienced DLTs requiring dose reductions, as specified by the protocol. Individual doses were delayed up to 4 days as specified in the protocol for neutropenia, with rapid resolution of the depressed neutrophil count. To address this, schedule B was created, with a planned time off treatment for 2 days of each week of therapy. With this change, the number of delayed doses was significantly decreased. None of the 15 schedule B patients experienced neutropenia DLTs requiring dose reductions.

DLTs were experienced in 4 patients: 3 from schedule A and 1 from schedule B (Table 2). One patient (Patient 01-012) on schedule A had 2 DLTs during the study. On schedule A, a severe neurologic DLT occurred in Patient 01-012 in cycle 1 at a dose of 0.80 mg/m^2^, and the prior dose level of 0.67 mg/m^2^ was expanded. The drug was discontinued after Patient 01-012 experienced grade 3 cerebellar toxicity considered related to Minnelide, which resolved without sequelae after drug discontinuation. At the 0.67 mg/m^2^ dose on schedule A, there were 2 hematologic DLTs (thrombocytopenia and neutropenia) of 6 treated patients during cycle 1. A grade 4 thrombocytopenia event was considered possibly related to Minnelide and the patient’s next dose was delayed; the event resolved. A grade 4 neutropenia event was considered definitely related to Minnelide and resulted in dose reduction, and a dose delay of 3 days; the event resolved. The MTD for schedule A was 0.53 mg/m^2^, due to the number of DLTs reported at the 0.67 mg/m^2^ dose level.

On schedule B, severe neurologic toxicity (grade 3 cerebellar dysfunction) was observed in 1 patient in cycle 1 at the dose level of 1.0 mg/m^2^, and the prior dose level of 0.8 mg/m^2^ was expanded to 6 patients without further DLT. The cerebellar dysfunction was considered related to Minnelide, study drug was discontinued, and the event resolved. The dose level of 0.8 mg/m^2^ was determined to be the MTD for schedule B dosing. The overall MTD of the study was 0.67 mg/m^2^.

**Figure 1.**
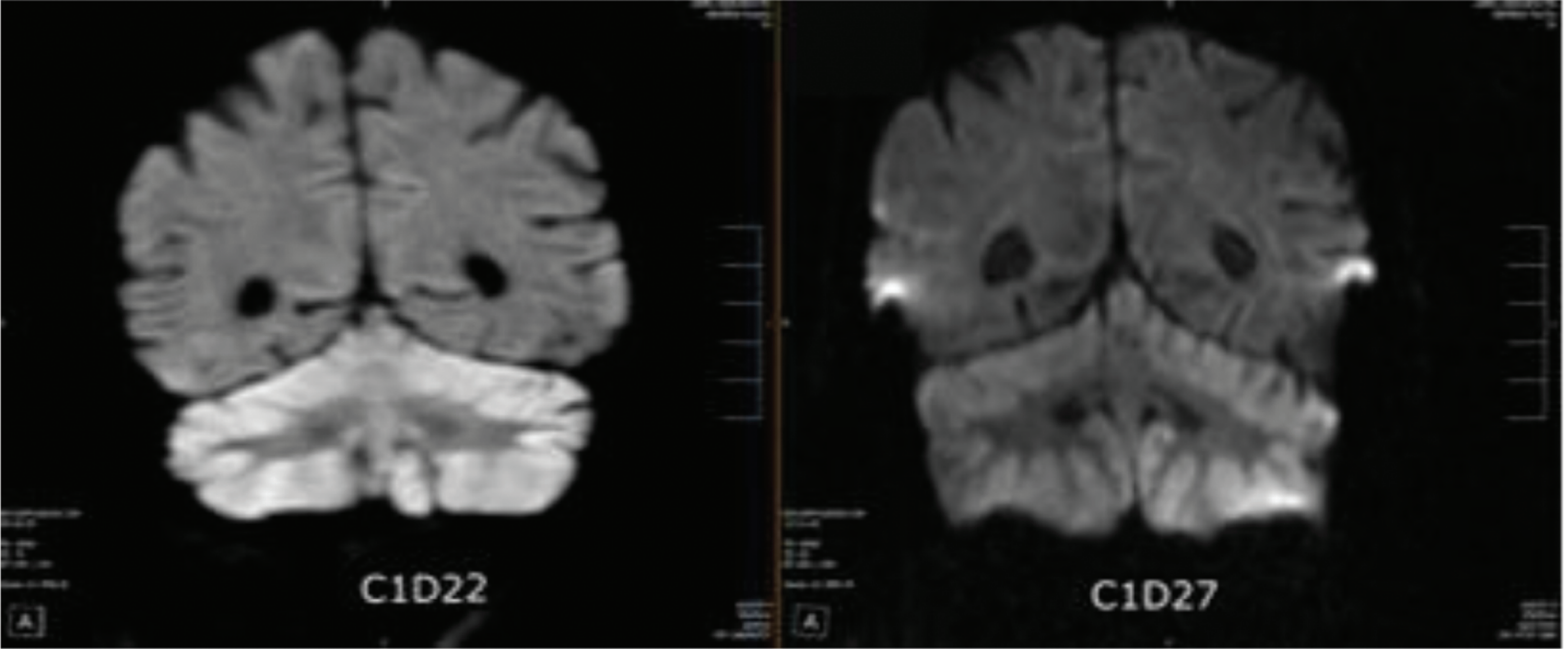
MRI brain of patient 012 with cerebellar toxicity of Minnelide. Marked cerebellar activity change is seen between days 22 (left) and 27 (right) of cycle 1.

Of the 2 patients who experienced severe but reversible cerebellar dysfunction DLTs, Patient 01-012 received 0.8 mg/m^2^ on schedule A, and Patient 01-112, 1.0 mg/m^2^ on schedule B. Both developed toxicity at the end of the third week of therapy during cycle 1. Onset was rapid, reaching maximum intensity within 48 hours of onset with severe ataxia and dysarthria, then resolving very slowly. Patient 01-012 died of progressive cancer within 6 weeks of onset, with significant improvement in the neurologic deficit at last evaluation 2 weeks prior to death. Patient 01-112 had complete resolution by 8 weeks after onset. MRI of the brain in both patients showed marked cerebellar changes (Patient 01-012 is shown in Fig. 1). Both patients demonstrated prolonged elevated blood levels of triptolide compared to other patients in the trial, described below.

Sixty-four percent of patients discontinued treatment for disease progression (27/42), 19% (8/42) stopped due to a TEAE, and 5% (2/42) withdrew consent. Of the discontinuations due to a TEAE 5% (2/42) were for treatment-related toxicity (DLTs) and 12% (5/42) were for non-treatment-related AEs. Six patients died within 30 days of therapy completion, with 5 deaths attributed to progression of underlying disease, with no recognized contribution of the study therapy to any death.

### Pharmacokinetics

PK samples were obtained from all patients except for one on schedule B. Following a single IV infusion dose of Minnelide, plasma concentrations reached C_max_ in all patients during or by the end of infusion (30 minutes) and were similar between patients on the same dose in schedules A and B (Supplementary Fig. S1). High interpatient PK variability was observed; therefore, interpretation was based on median (min-max) PK parameter estimates for each group. Minnelide was cleared rapidly from plasma, resulting in too few post-infusion datapoints to calculate T_1/2λz_ for most patients. Where calculable, half-life (T_1/2λz_) was ≤-0.165 hour (10 minutes) and the last quantifiable plasma concentrations occurred ≤1.0 hour. Minnelide plasma concentrations increased in a reasonably dose proportional manner. Minnelide PK were similar after multiple doses (day 15) as compared with day 1 in both dosing schedules. No accumulation was observed, which was consistent with the short half-life and the absence of predose Minnelide concentrations on days 8 and 15.

Minnelide was rapidly converted to triptolide in all patients, in both schedules, and at all dose levels, with peak triptolide levels observed shortly after completion of the 30-minute infusion (T_max_ range: 0.54 to 0.65 hour). The half-life of triptolide was approximately 1 hour (median: 0.76 hour; range: 0.36 to 2.3 hour), with complete clearance in most patients by 3 hours. Triptolide PK parameters C_max_ and AUC_last_ were similar and dose proportional in both dosing schedules following single Minnelide administration (Table 3). Triptolide PK appeared similar between days 1 and 15 among all dose groups (Supplementary Fig. S2).

**Table 3.** Triptolide pharmacokinetic parameters following single IV dose of Minnelide on cycle 1 day 1.

	Minnelide Dose (mg/m^2^)					
	0.16	0.32	0.53	0.67	0.80	1.00
Schedule A						
n	3	3	12	6	3	0
C_max_, median (min, max), ng/mL	4.09 (3.1, 5.1)	3.37 (2.9, 6.9)	7.82 (4.5, 14.8)	9.24 (5.0, 11.4)	12.50 (8.2, 13.7)	—
AUC_last,_ median (min, max), ng × hr/mL	3.47 (1.45, 5.31)	3.03 (2.81, 11.30)	7.50 (4.19, 13.60)	8.45 (5.85, 11.80)	11.4 (10.40, 33.80)	—
Schedule B						
n	0	0	0	5	6	4
C_max_, median (min, max), ng/mL	—	—	—	8.27 (7.5, 9.9)	10.75 (8.7, 15.7)	11.50 (9.8, 15.7)
AUC_last,_ median (min, max), ng × hr/mL	—	—	—	7.05 (6.77, 17.60)	10.33 (7.66, 16.30)	14.60 (12.20, 27.10)

In summary, Minnelide PK and triptolide PK were similar in patients from schedules A and B following a single (day 1) or multiple (days 1 and 15) doses. For both analytes, C_max_ occurred before or close to the end of infusion (≤30 minutes).

There were no notable differences between patients with and without serious AEs and/or DLT, except for 2 patients with severe cerebellar toxicity (Supplementary Fig. S2). These 2 patients exhibited similar Minnelide PKs and peak triptolide levels as compared to other patients at the same doses. However, the AUC_last_ for triptolide for each of these 2 patients was more than 2-fold greater than any other patient, consistent with delayed clearance of triptolide, with modestly higher levels for these 2 patients on day 15 than day 1.

### Efficacy

**Figure 2.**
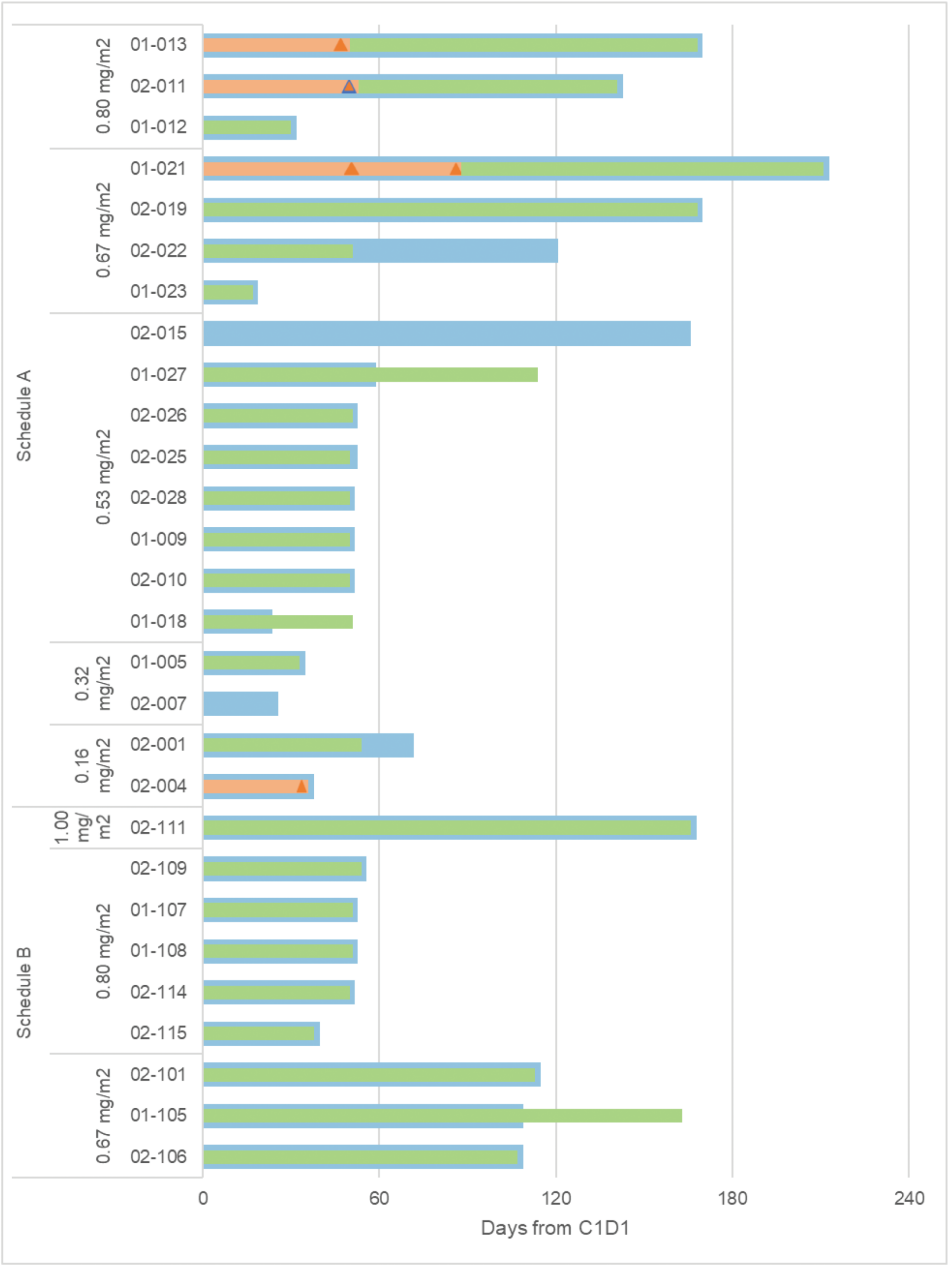
Response during the course of treatment in each patient. The green bars indicate the time to progression by Choi criteria, the blue bars indicate the time to progression by RECIST criteria, and the triangles indicate the time points when the patient met formal partial response criteria (orange for Choi and blue for RECIST).

Twenty-eight patients had sufficient imaging (measurable disease at baseline and at least one post-treatment scan) to allow measurement of response by RECIST criteria (Fig. 2). Central RECIST 1.1 assessments showed: 0/28 (0%) CR; 1/28 (4%) PR; 14/28 (50%) SD, and 13/28 (46%) PD events. The PR and SD event rates were 5% (1/19) and 47% (9/19) for schedule A and 0% (0/9) and 56% (5/9) for schedule B, respectively. Notably, SD was noted in 8 patients to cycle 2 (~2 months), 2 patients to cycle 4 (~4 months), 1 patient to cycle 5 (~5 months), and 2 patients up to cycle 6 (~6 months). One patient receiving 0.80 mg/m^2^ on schedule A showed PR at cycle 2 (~2 months) and SD at cycle 3 (~3 months).

Sixteen of 28 (57.1%) patients demonstrated a decrease in average target lesion tumor density by Choi criteria, and 12 of 28 patients had a greater than 15% change from baseline, suggesting some degree of target lesion necrosis (Supplementary Fig. S3). The mean best changes in target lesion average density from baseline were −35.7% for schedule A and −41.0% for schedule B.

**Figure 3.**
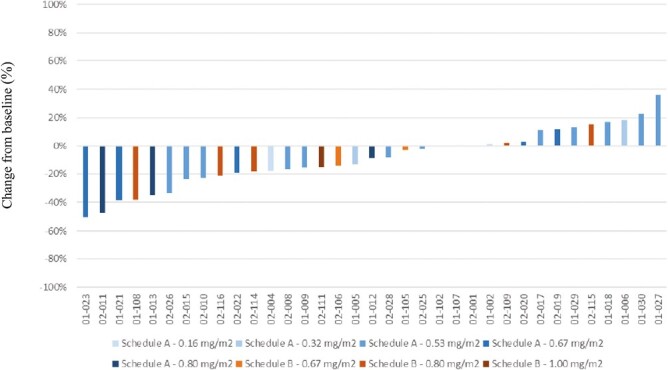
The best percent change in the sum of target lesion SUV_max_ from baseline per patient and dose level.

**Figure 4.**
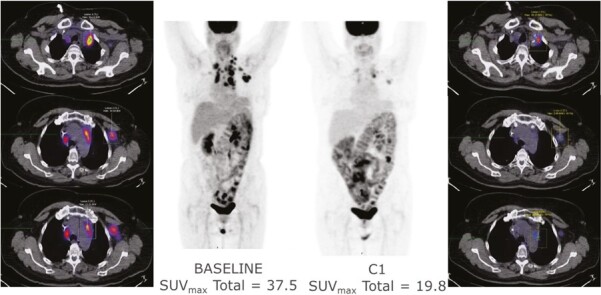
Best PET response partial metabolic response from gastric cancer patient 02-011.

Thirty-six patients had FDG PET imaging at baseline and 1 month for assessment of metabolic response by EORTC criteria. A waterfall plot depicts changes in SUV_max_ on PET imaging (Fig. 4). Of the 35 patients with FDG-avid disease, 23 (65.7%) demonstrated a decrease in SUV_max_ including 6 of 21 patients (17.1%) meeting the EORTC criteria for a partial metabolic response (>25% decrease in metabolic activity). Notably, one patient (02-011) with gastric cancer had a decrease in SUV_max_ from 37.5 at baseline to 19.8 (−47%) at cycle 1 and had an unconfirmed PR by RECIST (Figs. 3 and 4). There was no significant difference in median change in SUV_max_ between dosing schedules. The median reduction in SUV_max_ increased in a dose-dependent manner in both dosing schedules (data not shown).

The median PFS in schedule A for 19 patients was 57 days (95% CI, 33.0-164.0 days) and in schedule B for 9 patients was 107 days (95% CI, 38.0-113.0 days).

HSP70 levels had interpatient variability, however, generally decreased in schedule A and remained level in schedule B (Supplementary Fig. S4).

## Discussion

This first-in-human, phase I clinical study identified a safe dose and schedule of Minnelide in patients with advanced GI cancers for use in a subsequent study. The primary toxicity experienced was hematologic, in agreement with predictions from preclinical studies. However, there was a more rapid fall and recovery in neutrophil count than is typical of bone marrow toxic agents. This study was not designed to specifically address this phenomenon, but a marrow biopsy on 2 patients collected during neutropenia events did not have typical findings of marrow toxicity. Two patients also received steroids at the time of neutropenia without acute change in their neutropenia status. We speculate this may represent an alteration in trafficking or apoptosis of more mature hematologic cells. The neutropenia was improved by withholding treatment with Minnelide for 2 days each week (schedule B), allowing the escalation of the dose to a higher level. However, the alternative dosing schedule (schedule B) did not inhibit HSP70 levels to the same degree as schedule A.

The severe cerebellar toxicity experienced by 2 patients (also reported in Roshan^22^) was an unexpected finding as toxicity studies in animals found no neurologic toxicity. While not previously reported in peer-reviewed literature, there are reports of cerebellar toxicity from a succinate prodrug of triptolide in an abstract from a regional hematology meeting (personal communication from PI). The 2 patients with this toxicity had higher triptolide AUC_last_ than all other patients in this study, resulting in much greater drug exposure. Minnelide PK was unremarkable for these 2 patients, suggesting that clearance rather than formation of triptolide may be altered. The reason for this difference is uncertain because metabolism of triptolide is not well characterized. We did not identify any likely drug interactions or other common characteristic of these 2 patients that could explain the difference. The use of real-time measurement of plasma drug concentrations may allow future dose escalations of Minnelide while avoiding this toxicity.

While not a primary endpoint, this study provides preliminary evidence of efficacy of Minnelide treatment in this heavily pretreated, poor-prognosis group of patients. Several patients treated in this study experienced prolonged periods of disease control (SD) in circumstances in which rapid progression would have been expected. Stable disease ranged from approximately 2 to 6 months in 14/28 (50%) of evaluable patients, demonstrating clinical benefit. Notably, one patient showed PR at cycle 2 (~2 months) and SD at cycle 3 (~3 months).

In addition, Minnelide successfully targeted the intended mechanism of action by reducing plasma HSP70 levels. Although the sample size was small, the attenuated decrease in HSP70 levels observed in patients in schedule B suggests that withholding Minnelide therapy 2 days per week decreased the activity of the drug. And finally, the development of an oral formulation of Minnelide is being explored which has the potential to be more bearable as a schedule as opposed to an IV daily administration.

## Conclusion

In this study, Minnelide treatment exhibited some evidence of clinical activity in patients with refractory pancreatic and gastric cancer. Subsequent preclinical evaluation of Minnelide in gastric cancer cell lines and mouse xenograft models indicate a synergistic effect in combination with irinotecan.^23^ Synergistic activity with Minnelide and oxaliplatin has also been observed preclinically in pancreatic cancer models resistant to oxaliplatin.^24^ Combination therapeutic approaches with the oral formulation of Minnelide and protein-bound paclitaxel (abraxane) are currently being explored based on this early clinical experience and preclinical data (NCT03129139).^25^

The results of this study suggest that phase II studies of Minnelide should be conducted following a dosing schedule that includes daily Minnelide 0.67 mg/m^2^ treatment for 21 days in 28-day treatment cycles. Recently, a phase II trial following this schedule has completed accrual in individuals with refractory pancreatic cancer (NCT03117920).

## Supplementary Material

Supplementary material is available at *The Oncologist* online.

oyad278_suppl_Supplementary_Material

## Data Availability

The data underlying this article are available in the article and in its online supplementary material.
