# Supplementary material for: First-in-Human Phase I Study of Minnelide in Patients With Advanced Gastrointestinal Cancers: Safety, Pharmacokinetics, Pharmacodynamics, and Antitumor Activity
Source: Oncologist. 2024 Jan 3;29(2):132–41. doi: 10.1093/oncolo/oyad278 (PMC10836316; doi:10.1093/oncolo/oyad278)
Supplement: oyad278_suppl_Supplementary_Material [file oyad278_suppl_supplementary_material.docx]

Supplementary Information

**A**


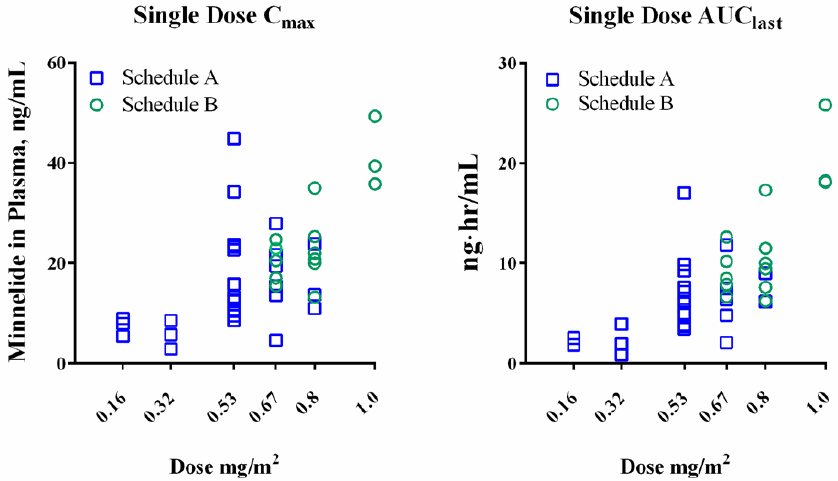


**B**


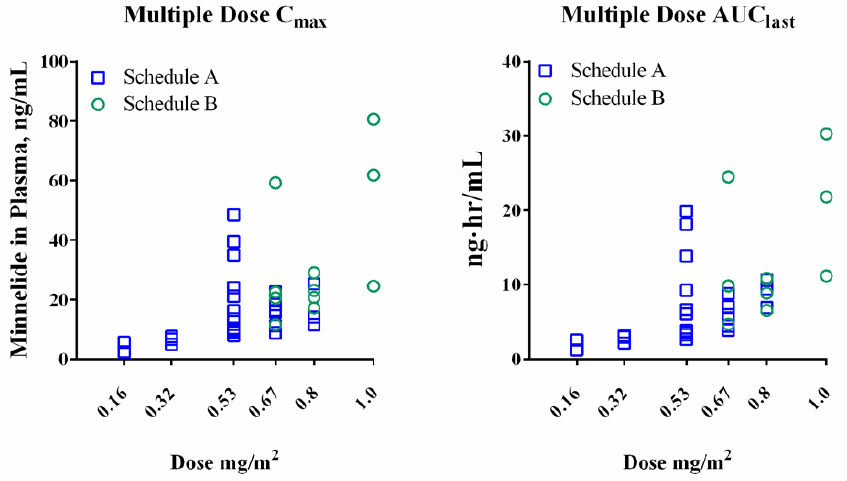


Supplementary Figure S1. Minnelide C_max_ and AUC_last_ versus dose following the first dose (A) and multiple doses (B).


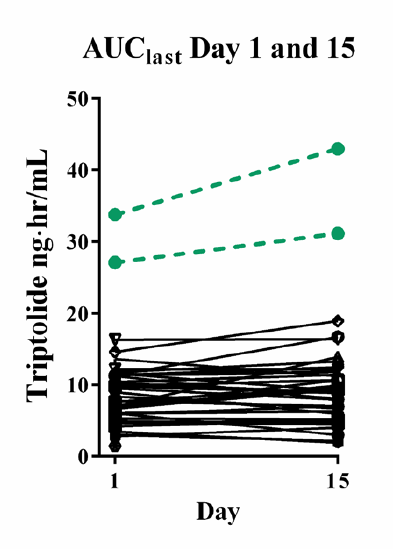


Supplementary Figure S2. Triptolide exposure (AUC_last_) for patients on days 1 and 15. Dashed green lines represent values in the two patients who developed severe cerebellar toxicity.


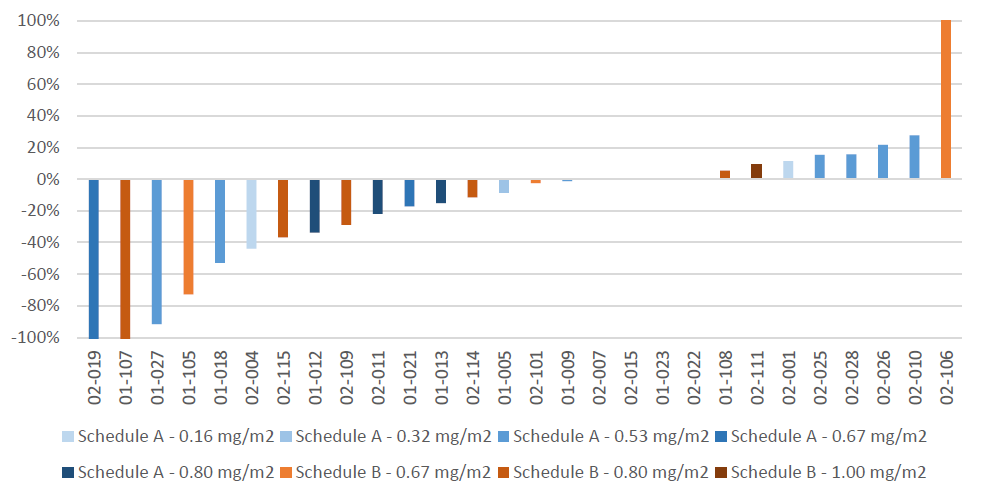


Change from baseline (%)

Supplementary Figure S3. The best percent change from baseline in average of target lesion density per dose level, as evaluated by Choi criteria. Please note that subjects 02-019, 01-107, and 02-106 had tumor density changes greater than 100% but not represented graphically in this figure.

Supplementary Figure S4. HSP70 response. Each plotted line indicates a plasma HSP70 concentration for an individual patient.
